# The Impact of Antibiotic Therapy Options and Multidisciplinary Approach in Prosthetic Joint Infections

**DOI:** 10.3390/microorganisms13102241

**Published:** 2025-09-24

**Authors:** João Lucas, José Queirós, Daniel Soares, André Carvalho, Filipa Pereira, Cláudia Santos, Ricardo Sousa, Miguel Araújo Abreu

**Affiliations:** 1 Orthopaedic and Traumatology Department, ULS do Alto Ave, 4835-044 Guimarães, Portugal; 2 Orthopaedic and Traumatology Department, ULS Santo António, 4099-001 Porto, Portugal; 3 Abel Salazar Institute of Biomedical Sciences, Porto University, 4050-313 Porto, Portugal; 4 Porto Bone and Joint Infection Group (GRIP), ULS Santo António, 4099-001 Porto, Portugal; 5 Hospital Lusíadas, Av. da Boavista 171, 4050-115 Porto, Portugal; 6 Infectious Diseases Department, ULS Santo António, 4099-001 Porto, Portugal

**Keywords:** anti-bacterial agents/therapeutic use, hip prosthesis, knee prosthesis, multidisciplinary team approach, prosthetic joint infections

## Abstract

Periprosthetic joint infection (PJI) remains one of the most challenging complications of arthroplasty. Optimal antibiotic strategies and the role of multidisciplinary teams (MDT) are not fully defined. We retrospectively analyzed 86 PJI surgical procedures performed between 2017 and 2023 at a tertiary referral center. Clinical data, microbiology, surgical strategy (debridement, antibiotics, and implant retention -DAIR, one-stage, two-stage) and antibiotic regimens were collected. Outcomes were compared across antibiotic classes and treatment teams: orthopaedics alone, orthopaedics with MDT input, and a dedicated MDT (GRIP). Success was defined as infection-free survival without further surgery. Median patient age was 70 years, with high comorbidity and predominance of Gram-positive, monomicrobial infections. Rifampicin-based regimens were associated with higher cure rates than non-anti-biofilm therapy (OR 4.9, 95% CI 1.4–17.8). Flucloxacillin plus rifampicin achieved outcomes comparable to rifampicin–fluoroquinolone combinations. The strongest predictor of success was MDT involvement: in DAIR procedures, cure reached 100% with MDT versus 48% with orthopaedics alone (*p* = 0.025). Outcomes were similar between teams in one- and two-stage revisions. In this cohort, rifampicin-based therapy improved outcomes in staphylococcal PJI, and flucloxacillin was a valid alternative partner drug. Crucially, MDT management—particularly in DAIR—was associated with superior results. These findings highlight the value of structured multidisciplinary PJI care pathways alongside optimised antibiotic strategies.

## 1. Introduction

Prosthetic joint infection (PJI) is a serious complication of hip and knee arthroplasty and a common cause for revision arthroplasty, representing a significant source of morbidity with profound negative effects on patients’ health status and quality of life. Rates of PJI in primary total hip and total knee arthroplasty range between 0.3% and 1.9%, and up to 10% in revision cases. Significant morbidity is associated with this devastating complication; the economic burden on our healthcare system is considerable, and the personal cost to the affected patient is immeasurable [1]. Correct management depends on the specific clinical scenario but usually combines some kind of surgery with implant retention or exchange revision, and appropriate antibiotics adapted to the causative microorganism(s), the patient and surgical strategy [2].

The hallmark of implant-related infections is the formation of biofilm on the implant surface, which significantly complicates the choice of appropriate antibiotic therapy. Biofilm-embedded bacteria exhibit reduced metabolic activity and altered phenotypes, making them less susceptible to antibiotics that are effective against planktonic organisms. Standard susceptibility testing does not reflect the higher concentrations required to eradicate biofilm-associated bacteria, leading to frequent clinical failures and recurrences [3,4,5]. Anti-biofilm activity is a critical determinant of treatment success, especially with an implant in place. For staphylococcal PJI, rifampicin is the cornerstone of anti-biofilm therapy due to its unique ability to penetrate biofilms, but it must always be combined with another active agent to prevent resistance. Doxycycline and daptomycin also demonstrate some anti-biofilm activity, but their efficacy is variable and generally inferior to rifampicin in this context. For Gram-negative organisms, fluoroquinolones are preferred for their biofilm activity, but rising resistance could limit their use [3,4,6,7].

Each case should be evaluated individually, with close multidisciplinary collaboration being critical for a successful outcome. As such, optimal management of PJI is best delivered in specialized centers by dedicated teams, including orthopedic surgeons and infectious disease specialists working together in a coordinated clinic. This approach is recommended due to the complexity and high cost of PJI treatment, the need for tailored management plans to address varying surgical circumstances and microbial causes, and the importance of unified decision-making for surgical and antimicrobial strategies [8].

The goal of this study is to assess the influence of different antibiotic regimens on the treatment success rate of PJI. In addition, we aimed to evaluate whether treating patients within or with the collaboration of our dedicated multidisciplinary team (MDT) (where antibiotic therapy was decided together with surgical strategy) had an influence on those success rates.

## 2. Materials and Methods

This is a retrospective study based on a prospectively maintained database, completed whenever necessary with review of electronic clinical records. Debridement, antibiotics and implant retention (DAIR) procedures, single or two-stage exchange surgery, performed for acute or chronic infections (with or without a prior DAIR procedure) were all considered.

Infection was defined according to the EBJIS definition. PJI is confirmed by the presence of a sinus tract, the isolation of identical organisms in at least two separate cultures, a total leukocyte count exceeding 3000 cells/μL or a polymorphonuclear neutrophil (PMN) proportion greater than 80%, growth of more than 50 CFU/mL from sonication fluid of any microorganism, or positive histopathological evidence of infection [9].

Patients who underwent surgery between January 2017 and December 2023 were included in the study. Clinical and demographic variables are collected into an institutional database, including age at revision surgery, sex, type and site of surgery, antibiotic therapy used, duration of antibiotic treatment, identified microorganisms, JS-BACH classification, and the outcome of the procedure. Minimum follow-up was 12 months. Patients with insufficient information or incomplete follow-up were excluded.

Concerning antibiotic regimens, our typical antibiotic treatment duration is 12 weeks for DAIR or One-stage cases. Two-stage exchange cases are mostly dealt with a 6-week course treatment after the first stage, followed by the second stage without antibiotic holidays. Duration of postoperative antibiotic treatment is 6 weeks if second-stage cultures are negative or 12 weeks if they are positive. Our standard empirical antibiotic therapy is vancomycin and piperacillin/tazobactam in all cases until culture results are available, and allows for targeted regimens. Such regimens were classified as “anti-biofilm” when an association including rifampicin was used for staphylococci and/or quinolones for Gram-negative infections. All patients initially received broad-spectrum empirical therapy, which was subsequently adapted according to microbiological results. Naturally, the susceptibility profile of the infecting microorganisms influenced the final antibiotic regimen; in some cases, resistance patterns limited the use of certain anti-biofilm options. Regarding the interval between diagnosis and surgery, these data were not consistently available in our retrospective cohort and could not be reliably analyzed. We acknowledge this as a limitation, as delays may affect clinical outcomes.

We also focused on the surgical team, which was responsible for the case and stratified patients according to the degree of multidisciplinary involvement in their care. In our institution we do have a multidisciplinary team (MDT) where a dedicated group of surgeons work alongside infectiologists, microbiologists, internal medicine specialists, etc. Patients treated by this team were managed through a structured and fully integrated protocol: all cases were reviewed in a dedicated weekly multidisciplinary team (MDT) meeting where treatment plans were defined collectively. This MDT process included decisions regarding diagnostic workup, surgical strategy, antibiotic therapy, timing of discharge and follow-up planning. Surgical procedures were then performed by dedicated surgeons, ensuring alignment between preoperative discussion and intraoperative execution.

Although this team exists, some cases are dealt with by arthroplasty surgeons, who are not necessarily experts in infected cases, and who may rely on MDT advice to help decide on surgical and antibiotic therapy strategy—we grouped them as “Ortho with MDT input” cases. Finally, some cases were managed exclusively by the orthopaedic team with no input from the MDT. In this subgroup, all surgical and medical treatment decisions (including antibiotic selection) were made independently by the treating orthopedic surgeon—we named them “Ortho alone” subgroup.

Continuous variables were expressed as mean ± standard deviation (SD) or as median with interquartile range (IQR), according to distribution, and compared using the Kruskal–Wallis test. Categorical variables were summarized as counts and percentages, and compared using the chi-square or Fisher’s exact test, as appropriate.

The primary outcome was treatment success, defined as infection-free survival without the need for further revision surgery. Univariable associations with outcome were first assessed using odds ratios (OR) with 95% confidence intervals (CI). Variables with *p* < 0.10 were subsequently entered into multivariable logistic regression to adjust for potential confounders (age, sex, ASA score, JS-Bach complexity index, comorbidities). Results are presented as adjusted OR with 95% CI.

Subgroup analyses were performed according to surgical procedure (DAIR, one-stage, two-stage) and antibiotic regimen (rifampicin-based versus non-anti-biofilm; flucloxacillin + rifampicin versus rifampicin + fluoroquinolone). A two-sided *p* < 0.05 was considered statistically significant. All analyses were performed using SPSS Statistics, version 23.0 (IBM Corp., Armonk, NY, USA).

## 3. Results

Ultimately, we were able to include 86 patients who underwent surgery for PJI in the study period, with an overall cure rate of 69.8% (60/86). The median age was 71 years (IQR 63–77). Most patients presented with significant comorbidity, as reflected by the fact that 62.8% were ASA class III or higher. They were divided into three groups according to the treatment team: 36 patients were managed by MDT, 17 Ortho with MDT input and 33 by the orthopedic team alone. Table 1 details the study population.

Regarding isolated microorganisms, Gram-positive microorganisms were isolated in the majority of cases (63.7%), and there was a predominance of monomicrobial infections (66.3%). Table 2 details the microorganisms identified in this study.

Treatment outcomes varied according to the causative microorganism. Among Gram-positive infections, the best results were observed in MSSA (87.5%), *S. lugdunensis* (80%), and *Enterococcus* spp. (77.8%), whereas *Streptococcus* spp. (71.4%) and CoNS (66.7%). MRSA presented the poorest outcome among staphylococci, with a cure rate of 60%. Gram-negative organisms were associated with worse prognosis overall, particularly Enterobacterales (54.5%). When stratified according to microbial pattern, monomicrobial infections were generally associated with higher treatment success compared with polymicrobial infections. Among the most frequent pathogens, success rates in monomicrobial cases were 91.7% for MSSA, 87.5% for *S. lugdunensis*, and 75.0% for CONS, whereas the corresponding outcomes in polymicrobial infections were 75.0%, 50.0%, and 57.1%, respectively. Statistical comparisons showed no significant differences, and analysis of antibiotic regimens within each pathogen group was similarly limited by the small sample size.

As shown in Table 3, no clinical factor reached statistical significance in the univariable analysis. However, trends were observed for several variables. Patients with higher ASA scores had poorer outcomes, with success rates decreasing from 100% in ASA I to 33% in ASA IV (*p* = 0.062). Similarly, the presence of at least one comorbidity (obesity, diabetes, or inflammatory arthritis) was associated with a lower success rate (61.8% vs. 75.0% in those without comorbidities, *p* = 0.137). Age, JS-BACH score, and polymicrobial infection were not associated with treatment failure. In the multivariable model, ASA remained the only variable retained, but it did not reach statistical significance.

The antibiotic regimen was an important determinant of treatment success in PJI. In the combined cohort of DAIR and one-stage exchange (procedures, treatment with anti-biofilm regimens was associated with higher rates of success compared with non-anti-biofilm therapy (89.3% vs. 66.7%), corresponding to an odds ratio (OR) of 4.17 (95% CI 0.67–26.02, *p* = 0.14). Rifampicin-containing regimens achieved 92.0% success versus 66.7% without rifampicin (OR 5.75, 95% CI 0.88–37.62, *p* = 0.073). Although neither comparison reached statistical significance, both showed a consistent trend in favor of anti-biofilm strategies.

When analyzed separately, DAIR procedures treated with anti-biofilm regimens had higher proportions of successful outcomes compared with those without, but the difference did not reach statistical significance. Similarly, in the one-stage subgroup, anti-biofilm regimens were associated with higher success than non-anti-biofilm therapy, though the effect estimates were imprecise and confidence intervals were wide due to the small sample size.

In staphylococcal infections, rifampicin-containing regimens were associated with significantly higher success compared with regimens without rifampicin (88.5% vs. 62.9%; OR 4.53, 95% CI 1.13–18.09; *p* = 0.038). Likewise, anti-biofilm therapy overall (rifampicin and/or fluoroquinolone) achieved significantly better outcomes than non-anti-biofilm regimens (87.5% vs. 58.6%; OR 4.94, 95% CI 1.37–17.81; *p* = 0.018)—Table 4. When restricted to DAIR and one-stage exchange procedures, rifampicin—particularly in combination with a fluoroquinolone or an active β-lactam—remained strongly associated with higher success, mirroring the significant effect observed in the overall cohort. Similarly, anti-biofilm therapy, in general, confirmed its global benefit and showed a consistent favorable trend within DAIR/One-stage, although statistical significance was not reached (*p* > 0.05), likely due to limited sample size.

Among staphylococcal infections treated with DAIR or one-stage exchange procedures, we compared the performance of different rifampicin-based combinations. Rifampicin plus fluoroquinolone achieved a success rate of 88.2% (15/17), while rifampicin plus flucloxacillin achieved 85.7% (6/7). The pairwise comparison yielded an odds ratio (OR) for success of 1.25 in favor of rifampicin plus fluoroquinolone (95% CI 0.09–16.50; *p* = 1.00), which remained non-significant after adjustment for ASA and JS-BACH (adjusted OR 1.82, 95% CI 0.11–30.59; *p* = 0.68). Rifampicin in combination with doxycycline or with trimethoprim-sulfamethoxazole was only rarely prescribed in this cohort, and the numbers were insufficient to derive reliable estimates; these results are summarized in Table 5.

Treatment success varied according to the type of surgical procedure: one-stage revision achieved the highest cure rate (88.2%), whereas two-stage revision and DAIR procedures showed comparable outcomes, with cure rates of 65.7% and 64.7%, respectively.

The degree of multidisciplinary involvement was also associated with outcome. Patients managed by the MDT or with MDT input achieved higher cure rates than those treated exclusively by orthopaedics (77.8% and 76.5% vs. 57.6%), although these differences did not reach statistical significance in the overall cohort.

Marked contrasts emerged within DAIR procedures, where MDT management achieved a 100% cure rate, compared with 47.6% in the orthopaedics-only group (*p* = 0.025), while intermediate results were seen with MDT input (83.3%). In one-stage revisions, cure rates were uniformly high across all groups (90–100%), with no significant differences, although interpretation is limited by the small number of cases treated solely by orthopaedics. In two-stage revisions (n = 35), outcomes were similar across groups: cure was achieved in 72.7% of patients managed by the MDT, 71.4% with MDT input, and 64.7% with orthopaedics alone, again without significant differences.

Multivariable analysis restricted to patients undergoing DAIR or one-stage revision—the procedures in which anti-biofilm antibiotic regimens are classically indicated—confirmed these findings. After adjustment for age, ASA score, JS-BACH classification, and polymicrobial infection, MDT management was associated with significantly higher odds of cure compared with orthopaedics alone (OR for cure 33.3, 95% CI 1.37→100, *p* = 0.031). MDT input also showed a trend toward improved outcomes (OR for cure 4.8, 95% CI 0.39–54.1, *p* = 0.221), but without statistical significance.

Analysis of case complexity yielded similar results. Among patients classified as ‘complex’ according to the JS-BACH scale, MDT management was associated with a 2.3-fold increase in the odds of cure compared with orthopaedics alone (OR 2.3, 95% CI 0.72–7.61, *p* = 0.164), while MDT input was associated with a 1.9-fold increase (OR 1.9, 95% CI 0.59–6.45, *p* = 0.281), though neither reached statistical significance. Comparable trends were observed when complex and limited-option cases were analyzed together (OR 2.5, *p* = 0.11). These findings are summarized in Table 6.

## 4. Discussion

Coagulase-negative staphylococci were the most frequently isolated pathogens in this cohort, with Gram-positive organisms accounting for nearly two thirds of cases and a predominance of monomicrobial infections [10]. These findings are consistent with the established epidemiology of PJI, where Staphylococcus aureus and coagulase-negative staphylococci are the leading causative agents [8,11]. In a large cohort study of 2067 hip and knee prosthetic joint infections (PJIs), coagulase-negative staphylococci (CoNS) accounted for 37% of isolates, making them the most common pathogen, followed by Staphylococcus aureus (24%) and other Gram-positive organisms; overall, aerobic Gram-positive bacteria represented 82% of cases. Monomicrobial infections comprised 70% of episodes, with polymicrobial infections at 25% and culture-negative cases at 5% [11]. Regarding outcomes, cure rates are generally higher in monomicrobial than in polymicrobial infections, although differences often do not reach statistical significance due to limited sample size and the multifactorial nature of PJI treatment outcomes [8].

The present study demonstrates that the choice of antibiotic regimen is a critical determinant of treatment success in PJI, with rifampicin-containing regimens in particular achieving statistically significant improvements in cure rates. In staphylococcal infections, rifampicin-based therapy resulted in 88.5% success compared with 62.9% without rifampicin (OR 4.53, 95% CI 1.13–18.09; *p* = 0.038), confirming the central role of this agent in biofilm-active strategies. When DAIR and one-stage exchange procedures were analyzed together, anti-biofilm regimens overall yielded higher success (89.3% vs. 66.7%; OR 4.17, 95% CI 0.67–26.02; *p* = 0.14), although this difference did not reach statistical significance, likely due to sample size. These findings are consistent with the pathophysiology of PJI, where biofilm formation on implant surfaces limits antibiotic penetration and fosters persistent infection. Systematic reviews and meta-analyses support these results, showing that adjunctive rifampicin reduces treatment failure, particularly in exchange arthroplasty, while its benefit in DAIR procedures appears more variable and may depend on timing, partner antibiotic, and pathogen susceptibility [8,12,13].

In our cohort of staphylococcal PJIs (MSSA, MRSA, and coagulase-negative staphylococci, including *S. lugdunensis*), rifampicin-containing regimens were associated with significantly higher cure rates compared with regimens without rifampicin (88.5% vs. 62.9%; OR 4.53, 95% CI 1.13–18.09; *p* = 0.038). Anti-biofilm therapy overall, defined as rifampicin and/or fluoroquinolone-based combinations, also achieved significantly better outcomes compared with non-anti-biofilm regimens (87.5% vs. 58.6%; OR 4.94, 95% CI 1.37–17.81; *p* = 0.018). These findings reflect the established understanding that biofilm formation on prosthetic material acts as a barrier to antibiotic activity and contributes to persistent infection. Systematic reviews and meta-analyses indicate that adjunctive rifampicin reduces treatment failure rates, especially in the context of exchange arthroplasty, though its benefit in DAIR procedures is more variable and may depend on timing, co-antibiotic selection, and pathogen susceptibility [8,12,13].

In patients with staphylococcal prosthetic joint infection treated with DAIR or one-stage exchange, our success rates were similar for rifampicin plus fluoroquinolone and rifampicin plus flucloxacillin, with no statistically significant difference in crude analysis or after adjustment for ASA and JS-BACH. The wide confidence intervals reflect the limited sample size, preventing definitive conclusions on superiority; nevertheless, our findings suggest comparable clinical performance between the two combinations when the organisms are susceptible.

This observation is consistent with the literature, which positions rifampicin as the cornerstone of anti-biofilm therapy in staphylococcal PJI. Reviews highlight fluoroquinolones as preferred partners because of excellent bone penetration, oral bioavailability, and biofilm activity [14], but they also acknowledge the role of anti-staphylococcal β-lactams (e.g., flucloxacillin/oxacillin) as valid alternatives when fluoroquinolones are contraindicated or undesirable. Our data reinforce this point: in MSSA infections, rifampicin plus flucloxacillin achieved outcomes comparable to rifampicin plus fluoroquinolone. Importantly, in our cohort, several staphylococcal isolates were oxacillin-susceptible but quinolone-resistant, underlining the need to individualize partner drug choice according to microbial susceptibility, comorbidities, drug interactions, and tolerability.”

Our study also demonstrates that the degree of MDT involvement was associated with improved treatment outcomes in PJI, particularly in procedures where anti-biofilm strategies are most relevant. Overall, patients managed by or with input from the MDT achieved higher cure rates than those treated exclusively by orthopaedics (77.8% and 76.5% vs. 57.6%), although these differences did not reach statistical significance in the full cohort. Importantly, within the DAIR subgroup, MDT management was strongly associated with superior outcomes, achieving a 100% cure rate compared with only 47.6% in orthopaedics-only cases (*p* = 0.025), while MDT input achieved intermediate results (83.3%). These findings were confirmed by multivariable analysis, in which MDT management conferred a >30-fold increase in the odds of cure compared with orthopaedics alone, independent of patient- and infection-related factors.

These findings suggest a dose–response-like effect of multidisciplinary involvement: outcomes improved progressively from general orthopaedics alone, through partial MDT input, to full MDT management. Although intermediate cases did not reach statistical significance, the upward trend supports the hypothesis of an additive benefit from structured multidisciplinary input. The superior results observed with MDT management in DAIR procedures likely reflect not only optimized antibiotic selection—tailored with microbiology input—but also timely surgical decision-making, standardized follow-up, and coordinated discharge planning. These findings may also, at least in part, be attributable to the greater routine and surgical expertise of the dedicated team in managing PJI cases, which could have independently contributed to improved outcomes. In one-stage revisions, cure rates were uniformly high (90–100%) across all groups; however, no meaningful conclusions can be drawn regarding the effect of MDT involvement in this setting, as only two patients underwent one-stage exchange managed exclusively by orthopaedics. In two-stage revisions, outcomes were similar across management strategies, most likely due to the small number of cases per subgroup; nevertheless, a non-significant trend still favored MDT care.

Our findings align with recent guidelines and consensus statements, which strongly advocate for MDT-based PJI management as the standard of care [8].

## 5. Conclusions

In this cohort of PJI revisions, rifampicin-based therapy was associated with higher cure rates, and flucloxacillin performed comparably to fluoroquinolones as a rifampicin partner in staphylococcal infections. Crucially, multidisciplinary team management was the strongest determinant of success, particularly in DAIR procedures. These findings support the implementation of structured MDT involvement alongside optimised antibiotic strategies in the management of PJI. Beyond our institution, the results highlight the potential value of establishing formal multidisciplinary PJI management protocols in other hospitals, ensuring that antibiotic decisions, surgical planning, and follow-up are made in an integrated and coordinated manner. Our dedicated team is also available to support surrounding hospitals, which frequently request assistance in complex infection cases, and this collaborative network may further facilitate the dissemination and adoption of structured PJI care pathways.

## Figures and Tables

**Table 1:** Patient characteristics stratified by treatment team.

	MDT	MDT Input	Orthopedic Team
	n = 36	n = 17	n = 33
Mean age (±SD)	67.1 ± 11.8	69.4 ± 8.8	71.7 ± 11.1
Female gender	21 (58.3%)	8 (47.1%)	19 (57.6%)
Knee location	24 (66.7%)	8 (47.1%)	10 (30.3%)
Procedure			
DAIR	7 (19.4%)	6 (35.3%)	21 (63.6%)
One-stage	10 (27.8%)	4 (23.5%)	3 (9.1%)
Two-stage	19 (52.8%)	7 (41.2%)	9 (27.3%)
Comorbidities			
Obesity (BMI ≥ 30)	7 (19.4%)	4 (23.5%)	9 (27.3%)
Diabetes	13 (36.1%)	4 (23.5%)	9 (27.3%)
Inflammatory Arthritis	1 (2.8%)	2 (11.8%)	-
ASA ≥ 3	18 (50%)	14 (82.4%)	22 (66.7%)
JS BACH			
Uncomplicated	13 (36.1%)	-	16 (48.5%)
Complicated	20 (55.6%)	13 (76.4%)	16 (48.5%)
Limited options	3 (8.3%)	4 (23.5%)	1 (3%)

**Table 2:** Detailed description of the identified pathogens in the study population.

Isolated Pathogens	Total	Cure Rate
*Staphylococcus aureus*	*21*	81%
*MRSA*	5	*60%*
*MSSA*	16	87.5%
*Coagulase-Negative Staphylococci (CoNS)*	*40*	70%
*Coagulase-Negative Staphylococci (excluding S. lugdunensis)*	*30*	66.7%
*S. lugdunensis*	*10*	80%
*Streptococcus* spp	*7*	*71.4%*
*Enterococcus* spp.	*9*	77.8%
*Cutibacterium acnes*	*2*	50%
*Enterobacterales*	11	*54.5%*
*Pseudomonas* spp.	*7*	*71.4%*
*Others*	25	56%
*Culture-negative*	2	50%

**Table 3:** Clinical predictors of treatment outcome.

Variable	Univariable OR (95% CI)	*p*-Value	Multivariable OR (95% CI)	*p*-Value
Age (per year increase)	1.02 (0.99–1.06)	0.155	-	-
ASA score (I–IV)	(trend across categories)	0.062	1.32 (0.87–2.14)	0.132
JS-BACH	1.15 (0.87–1.53)	0.273	-	-
≥1 comorbidity (obesity, diabetes, inflammatory arthritis)	1.83 (0.82–4.07)	0.137	-	-
Polymicrobial infection (Yes vs. No)	0.81 (0.33–1.97)	0.622	-	-

**Table 4:** Association of biofilm-active antibiotic regimens with treatment success in staphylococcal prosthetic joint infections.

	With Biofilm-Active Therapy (n)	Success (%)	Without Biofilm-Active Therapy (n)	Success (%)	OR	95% CI	*p*-Value
Rifampicin vs. No rifampicin	26	88.5%	35	62.9%	4.53	1.13–18.09	0.038
Anti-biofilm vs. No anti-biofilm (rifampicin and/or fluoroquinolone)	32	87.5%	29	58.6%	4.94	1.37–17.81	0.018

**Table 5:** Outcomes of rifampicin-based antibiotic combinations against staphylococcal infections in DAIR and one-stage exchange procedures.

	Regimen	N	Success (%)	95% CI	*p*-Value
One-stage + DAIR	Rifampicin + FQ	17	88.2%		
	Flucloxacillin + Rifamp.	7	85.7%	0.09–16.50	1

**Table 6:** Treatment outcomes according to team involvement.

	MDT	MDT Input	Orthopedic Team
	n = 36	n = 17	n = 33
Mean age (±SD)	67.1 ± 11.8	69.4 ± 8.8	71.7 ± 11.1
ASA Score	2.5 ± 0.7	2.9 ± 0.6	2.7 ± 0.7
Cure Rate			
DAIR	7/7 (100%)	5/6 (83.3%)	10/21 (47.6%)
One-stage	9/10 (90%)	3/4 (75%)	3/3 (100%)
Two-stage	12/19 (63.2%)	5/7 (71.4%)	6/9 (66.7%)
Cure rate (overall)	77.8%	76.5%	57.6%
Adjusted OR vs. Ortho alone [p] (overall)	2.58 [0.12]	2.4 [0.23]	1 (ref)
Adjusted OR vs. Ortho alone [p] (DAIR + 1T)	13.54 [0.019]	3.38 [0.18]	1 (ref)
Complex cases (JS-BACH)	20 (55.6%)	13 (76.5%)	16 (48.5%)
Adjusted OR vs. Ortho alone [p] (Complex cases)	2.3 [0.164]	1.9 [0.281]	1 (ref)

## Data Availability

The data presented in this study are available on request from the corresponding author. The data are not publicly available due to privacy and ethical restrictions.

## Abbreviations

ASA	American Society of Anesthesiologists (score)
CI	Confidence Interval
CoNS	Coagulase-Negative Staphylococci
DAIR	Debridement, Antibiotics, and Implant Retention
EBJIS	European Bone and Joint Infection Society
FQ	Fluoroquinolone
GRIP	Porto Bone and Joint Infection Group
IQR	Interquartile Range
JS-BACH	Joint-Specific BACH classification
MDT	Multidisciplinary Team
MRSA	Methicillin-Resistant *Staphylococcus aureus*
MSSA	Methicillin-Susceptible *Staphylococcus aureus*
OR	Odds Ratio
PJI	Prosthetic Joint Infection
PMN	Polymorphonuclear Neutrophil
R1T	One-stage Revision (single-stage exchange)
R2T	Two-stage Revision (two-stage exchange)
SD	Standard Deviation
